# Hypothyroidism: playing the cardiometabolic risk concerto

**DOI:** 10.1186/s13044-025-00233-y

**Published:** 2025-05-20

**Authors:** George J. Kahaly, Youshuo Liu, Luca Persani

**Affiliations:** 1 Department of Medicine I, Johannes Gutenberg University (JGU) Medical Center, Mainz, DE–55101 Germany; 2 Department of Geriatrics and Geriatric Endocrinology, Institute of Aging and Age-Related Disease Research, The Second Xiangya Hospital of Central South University, Central South University, Changsha, Hunan China; 3 Department of Medical Biotechnology and Translational Medicine, University of Milan, Milan, Italy; 4 Department of Endocrine and Metabolic Diseases, IRCCS Istituto Auxologico Italiano, Milan, Italy

**Keywords:** Hypothyroidism, Cardiometabolic risk factors, Cardiometabolic outcomes, Levothyroxine, Triiodothyronine, Drug safety

## Abstract

**Background:**

Thyroid hormones influence the function of essentially every system of the body, including the cardiovascular and metabolic system. Thyroid hormone replacement with levothyroxine (LT4) is the mainstay of pharmacological management for people with (especially clinically overt) hypothyroidism, and it is important to ensure the cardiovascular and metabolic safety of this treatment. This is especially so as in hypothyroidism, cardiometabolic risk factors and cardiovascular disease are highly prevalent conditions and will often coexist in an individual patient. Accordingly, we have reviewed the cardiometabolic consequences of hypothyroidism and intervention with thyroid hormone replacement.

**Main body:**

Numerous observational studies and meta-analyses have described multiple potentially adverse cardiometabolic consequences of hypothyroidism, including exacerbation of cardiovascular and metabolic risk factors (especially dyslipidaemia), functional impairment of the heart and vasculature (including accelerated atherosclerosis) and increased risk of advanced cardiovascular outcomes. LT4 usually improves cardiometabolic risk factors in people with hypothyroidism and some (but not all) studies have reported improved vascular and cardiac function in LT4-treated populations. Observational data have suggested the possibility of improved cardiometabolic outcomes with LT4 treatment, particularly in younger people with hypothyroidism, although data from randomised, controlled trials are needed here. Importantly, LT4 (with or without additional triiodothyronine) appears to be safe from a cardiovascular perspective, as long as overtreatment and iatrogenic thyrotoxicosis are avoided.

**Conclusions:**

Overall, the current evidence base supports intervention with LT4 to protect the cardiometabolic health of people with hypothyroidism who require thyroid hormone replacement, although more data on long-term clinical outcomes are needed.

## Introduction

As many as about 10% of all adults worldwide demonstrate features of clinically overt or subclinical hypothyroidism (half of which may be undiagnosed), with generally higher prevalence findings as age increases [1, 2]. The condition presents either as subclinical hypothyroidism (SCH), where thyrotropin (thyroid stimulating hormone, TSH) is elevated but free thyroxine (fT4) remains within the normal range, or the more severe, overt hypothyroidism (OH), where TSH is elevated and the level of fT4 is depressed [3]. As a prevalent and (usually) lifelong condition, considerable overlap will be found between populations with hypothyroidism and other common conditions [4]. Accordingly, it is important to note that the simultaneous occurrence of hypothyroidism (including that requiring intervention with LT4) and cardiometabolic risk factors or established cardiovascular disease is inevitably a common clinical finding.

We have known for decades that both the condition of hypothyroidism itself, and its principal pharmacologic treatment, levothyroxine (LT4), can exert a potentially profound impact on the function of the cardiovascular and metabolic system [5–7]. Cardiovascular disease is the leading cause of death worldwide [8, 9], so it is important, therefore, to optimise the cardiovascular and metabolic safety of intervention with LT4 in people with hypothyroidism, from the dual perspectives of minimising any adverse cardiovascular effects of thyroid dysfunction per se, while avoiding any possibility of new or exacerbation of cardiometabolic risk associated with its treatment [10, 11]. Here, we review the latest findings from the clinical literature on the impact of hypothyroidism and its treatment on cardiometabolic risk factors, and on clinical cardiovascular outcomes.

## Our approach to this review

As described above, the purpose of our review is to consider the impacts of OH and SCH, and of intervention with LT4 to manage hypothyroidism, on cardiometabolic risk factors and on clinical cardiovascular outcomes. The clinical literature on hypothyroidism is vast. For conciseness and clarity, we have considered more important observational studies alongside meta-analyses and randomised, controlled trials for effects on the various cardiometabolic risk factors and cardiovascular outcomes reviewed below. We also include a brief summary of the clinical implications of the current evidence base for each risk factor, to aid the reader in navigating this complex clinical landscape.

## Hypothyroidism and classical cardiometabolic risk factors

**Fig. 1:**
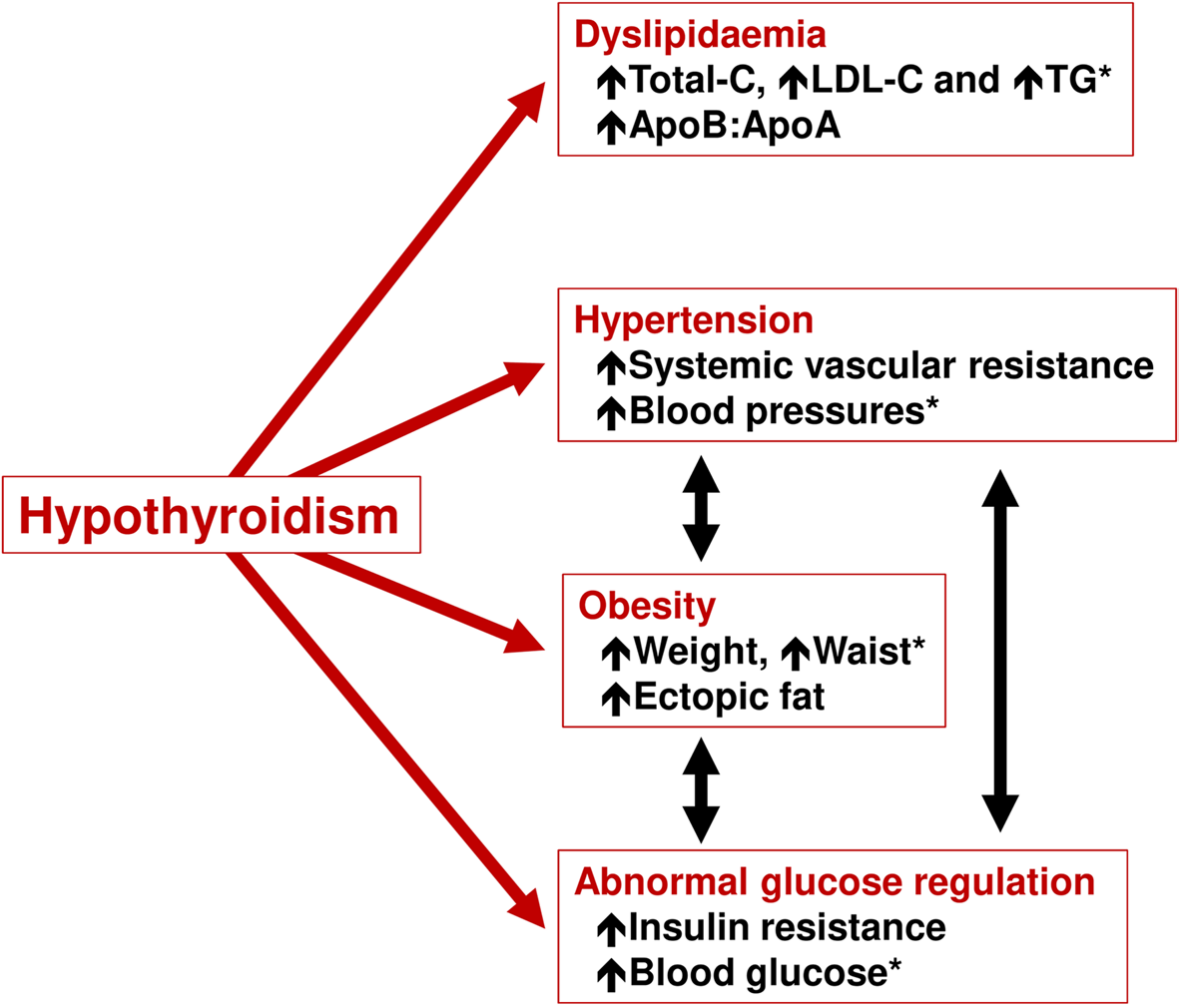
Overview of the effects of hypothyroidism on classical cardiometabolic risk factors: note that all of these are often improved by treatment with levothyroxine *Components of the metabolic syndrome. In general, the severity of disturbances of these risk factors varies with the severity of hypothyroidism: thus, these associations maty be considered to be more established for overt hypothyroidism, although clinical studies have also described them in people with subclinical hypothyroidism (see text for further explanation and supporting references)

Table 1 contains a summary of studies that associated hypothyroidism with classical cardiometabolic risk factors. These are also summarised in Fig. 1.

**Table 1:** Overview of studies of cardiovascular risk factors in people with hypothyroidism

Ref	Type	Main findings
**Hypertension**		
[12, 13]	Obs Obs	Increased diastolic and/or systolic BP or both for populations with OH or SCH vs. euthyroidism
[14]	Obs	Mendelian randomisation study showed that genetically predicted low T4 predicted hypertension
[﻿^15^, ^16^]	Obs	High TSH or low T4 predicted hypertension
[17]	Obs	No significant association between SCH and hypertension or BP
[18–20]	MA	Meta-analyses associated SCH with increased risk of hypertension of variable magnitude
**Dyslipidaemia**		
[14]	Obs	Mendelian randomisation study showed that genetically predicted low T4 predicted dyslipidaemia
[21]	Obs	Positive correlation between serum TSH and total-C, LDL-C and Apolipoprotein B
[22]	Obs	The lipid profile in SCH may be more atherogenic (increased lipid peroxidation)
[23]	Obs	No significant association of OH or SCH with dyslipidaemia after adjustment for other factors
[24–28]	MA	Meta-analyses confirmed significant associations of SCH with adverse lipid profiles
**Impaired blood glucose control**		
[14]	Obs	Mendelian randomisation study showed that genetically predicted low T4 predicted dyslipidaemia
[29, 30]	Obs	OH or SCH was associated with insulin resistance, diminished insulin secretion and increased hepatic glucose production
[31, 32]	Obs	High-normal TSH levels or low-normal T4 (within reference ranges) predicted development of type 2 diabetes or prediabetes, respectively
[33, 34]	Obs	Increased prevalence of diabetes in people with hypothyroidism
[35]	Obs	Thyroid function was depressed in people with vs. without new-onset diabetes
[36]	Obs	More diabetes-associated hypoglycaemia in people with vs. without thyroid disease (unspecified)
**Metabolic syndrome**		
[37]	Obs	No overall increase in metabolic syndrome prevalence with SCH, but some components were more common
[38]	Obs	Increased prevalence of metabolic syndrome in people with SCH vs. euthyroidism
[39]	Obs	Increased risk of hypothyroidism in people with metabolic syndrome
[20, 37, 38, 40]	MA	Increased prevalence of metabolic syndrome associated with SCH according to several diagnostic criteria (not criteria from China in one analysis)
[41]	SR	Thyroid function markedly influences all components of the metabolic syndrome
**Obesity**		
[42]	Obs	About three-quarters of people with OH or SCH are overweight or obese
[43, 44]	Obs	Obese subjects are at increased risk of thyroid autoimmunity

Study types: *Obs* observational study, *MA* meta-analysis. Other abbreviations: *BP* blood pressure, *T4* thyroxine, *OH* overt hypothyroidism, *SCH* subclinical hypothyroidism, *TSH* thyrotropin

### Blood pressure

#### Impact of hypothyroidism

Hypothyroidism increases systemic vascular resistance, with decreased cardiac preload and increased afterload [45]. Observational studies have demonstrated increased diastolic blood pressure (DBP), or both diastolic and systolic blood pressure (SBP), in people with hypothyroidism vs. euthyroidism [12, 13], including in a Mendelian randomisation study [14]. Conversely, people with high TSH were more likely to have hypertension in one study [15]. Measures of blood pressure (BP) have been shown to correlate significantly with markers of thyroid function, such as T4 or TSH [12, 15, 16], although variation in TSH did not explain a higher prevalence of hypertension in people with SCH [17]. Observational data have not always reported marked associations between hypothyroidism and increased BP in people with SCH [46]. Long-term data from 621 LT4-treated subjects in Brazil showed that the use of antihypertensive treatment increased over time, indicating that periodic review of BP and other cardiovascular risk factors is needed for this population [47].

A recent meta-analysis described significant elevations of BP in middle-aged women with SCH (odds ratio [OR] 1.64 [95%CI 1.18 to 2.27]), while there was no significant association in older women (OR 0.97 [0.80 to 1.16]) [18]. Another such analysis, of observational studies, reported only a small average difference in BP of 1.5/0.4 mmHg between populations with SCH and euthyroidism [19]. An analysis of the effects of hypothyroidism on the incidence of the metabolic syndrome (described below) also found an increase in BP in the hypothyroid group [20].

#### Impact of LT4

A reduction in DBP (− 2.6 mmHg [95%CI –0.4 to –4.8], p = 0.021) was observed in 60 patients with OH or SCH who were prescribed LT4 and followed for 12 weeks [48]. LT4 treatment reduced BP in an observational study in 30 women with elevated TSH vs. healthy control subjects [49]. In another study, an increase in DBP was observed in a population who underwent sudden, severe hypothyroidism due to withdrawal of LT4 treatment following total thyroidectomy, which suggests opposite influences of hypothyroidism per se and LT4 on BP [50].

A recent (2024) meta-analysis of 34 studies showed that intervention with LT4 for SCH was associated with an average reduction (95%CI) in BP of − 4.0 (–4.6 to –6.5)/ − 2.1 (–0.6 to –3.7) mmHg (both p < 0.05) [51]. An earlier analysis (2018) reported that average reductions (95%CI) in BP associated with LT4 treatment were − 2.5 (–0.3 to –4.6)/ − 0/9 (–2.3 to 0.6) mmHg (p = 0.024/p = NS) in patients enrolled in randomised trials and − 4.8 (–3.1 to –6.5)/ − 2.7 (–1.4 to –4.1) mmHg (p < 0.001 for each) [52]. Another meta-analysis, which focussed on older people with SCH, found no effect of LT4 on BP, although other cardiovascular risk factors improved (see below) [53].

Hypothyroidism and hypertension: summary of clinical implications The association of hypothyroidism with increased blood pressure has not been a universal finding (especially for SBP), although multiple observational and meta-analytic studies have reported such a finding. In addition, meta-analyses have suggested that intervention with LT4 reduces BP to a clinically significant extent in people with hypothyroidism. The observation in one study that effects of LT4 on BP were larger in observational studies than randomised trials suggests that the former may overestimate the contribution of reduced BP to overall cardiovascular risk management on this population.

### Dyslipidaemia

#### Impact of hypothyroidism

Clinically significant hyperlipidaemia, with increases in ApoB-containing lipoproteins (e.g. total- and low-density lipoprotein cholesterol [LDL-C]) and triglycerides, is a well-recognised biochemical consequence of hypothyroidism, including SCH [1, 3, 54–56]. Effects on high density lipoprotein (HDL-C) are variable, although there is usually an increase in the ratio of ApoB:ApoA-containing lipoproteins. [56] Disturbances of multiple pathophysiological processes contribute to the effects of hypothyroidism on the lipid profile, including increased cholesterol production (e.g. via inhibition of hepatic HMG-CoA reductase and increased intestinal cholesterol absorption) and reduced clearance of cholesterol (e.g. via reduced density of hepatic LDL receptors), as well as other processes that contribute to an atherogenic lipid profile, including inhibition of reverse cholesterol transport [54–58]. Genetically predicted low T4 was associated significantly with dyslipidaemia [14]. Serum TSH has been shown to correlate positively with total cholesterol (total-C), LDL-cholesterol (LDL-C) and ApoB [21]. Increased lipid peroxidation in people with SCH may indicate a more atherogenic lipid profile in this population [22].

Meta-analyses found adverse lipid profiles in people with SCH, including in populations with comorbid polycystic ovary syndrome, a strongly insulin resistant state [20, 24–28]. Overall, it has been estimated that as many as 90% of people with OH have dyslipidaemia [57]. Not all studies have associated hypothyroidism with dyslipidaemia after adjustment for other factors, however [23].

#### Impact of LT4

Randomised, placebo-controlled trials have demonstrated modest, but clinically significant, reductions in indices of hyperlipidaemia, including total-C and LDL-C, in people with SCH after treatment with LT4 [59–63].Observational data also supported a reduction of total-C, LDL-C, HDL-C, VLDL-C and triglycerides after LT4 treatment in people with OH or SCH [21, 22, 48, 64–68]. Meta-analysis of studies conducted in people with SCH found that treatment with LT4 improved multiple aspects of the lipid profile (total-C, LDL-C, triglycerides and ApoB) [53, 69].

Hypothyroidism has also been associated with increased deposition of ectopic fat in the liver [57], which is a strong predictor of future dyslipidaemia, hyperglycaemia, metabolic-dysfunction-associated fatty liver disease (MAFLD) and increased cardiometabolic risk [70, 71]. LT4 treatment reduced liver lipid content in an observational study in euthyroid people with type 2 diabetes and MAFLD. While comparable data from populations with a diagnosis of hypothyroidism are awaited, MAFLD has been described as a state of "intrahepatic hypothyroidism" [72]. Treatment with LT4 also reduced ectopic fat in the heart in a population with SCH [73].

Hypothyroidism and dyslipidaemia: summary of clinical implications Clinically significant dyslipidaemia is a well-known complication of hypothyroidism, and a substantial evidence base of clinical data supports improvement of the lipid profile following thyroid hormone replacement with LT4. However, the disturbances of the lipid profile associated with SCH may be relatively mild, and the effects of LT4 on lipids may be relatively modest. In addition, dyslipidaemia is a common condition and may occur independently in parallel with hypothyroidism. Care should be taken to identify and correct an abnormal lipid profile with standard lipid modifying therapy, according to prevailing cardiovascular management guidelines, in addition to correction of thyroid hormone levels with LT4, where indicated clinically.

### Blood glucose control

#### Impact of hypothyroidism

Insulin resistance appears to accompany hypothyroidism [29], with diminished pancreatic insulin secretion and increased hepatic glucose production, both of which are likely to predispose the patient to the development of fasting hyperglycaemia [30]. A prospective cohort study showed that higher TSH predicted a higher risk of developing type 2 diabetes, even within the reference range for this parameter [31]. Low-normal FT4 has been shown elsewhere to predict the presence of prediabetes [32]. Several other studies (reviewed elsewhere) have described epidemiological associations between the presence of diabetes and hypothyroidism [14, 33, 34]. A cross-sectional study in hospitalised patients showed that FT4, triiodothyronine (T3) and TSH were lower in people with vs. without new-onset diabetes [35]. Thyroid dysfunction in general has been shown to increase the risk of hypoglycaemia [36], particularly in the context of rare autoimmune disturbances of insulin action such as insulin autoimmune syndrome (Hirata's disease) or type B insulin resistance syndrome [74, 75].

A meta-analysis found no increase in the risk of incident diabetes in people with SCH [76], although others found that the presence of diabetes significantly predicted the presence of comorbid SCH [77] and TSH above its reference range predicted a 26% increase in the risk of diabetes [78]. Two additional meta-analyses have described greater severity of diabetes complications in people with SCH and diabetes, especially in the microvasculature [77, 79].

#### Impact of LT4

No randomised, controlled trials of LT4 are available from populations with type 2 diabetes and hypothyroidism. Observational data from populations with SCH [66, 67] or more severe hypothyroidism [48, 80] have suggested improvements in blood glucose and/or indices or insulin resistance; although no effect on insulin resistance was seen in another study in patients with primary hypothyroidism [64]. Another observational study suggested that LT4 had little effect on HbA1c in people without diabetes [81]. Finally, a systematic review reported a reduced rate of progression of diabetic nephropathy associated with LT4 treatment vs. placebo, although long-term data were lacking [82].

Hypothyroidism and impaired glycaemic control: summary of clinical implications There appear to be epidemiologic and pathophysiological connections between hypothyroidism and disturbances of blood glucose control, although these seem to be limited in extent and not seen universally in clinical studies. The meta-analytic evidence of improved renal outcome in people with diabetes who received LT4 is intriguing, and more randomised, controlled trials are urgently needed from this population. Hypothyroidism is treated with LT4, and LT4 may reduce the hypoglycaemic effect of antidiabetic drugs: therefore, patients' blood glucose levels should be frequently monitored, and if necessary, the dosage of anti-diabetic medications should be adjusted when initiating or adjusting LT4 therapy.

### Metabolic syndrome

#### Impact of hypothyroidism

The metabolic syndrome comprises a constellation of five cardiometabolic risk factors (hypertension, hyperglycaemia, hypertriglyceridaemia, low HDL-C, and abdominal obesity) that are associated closely and pathophysiological with insulin resistance [83]. There is no doubt that the presence of the metabolic syndrome confers increased risk of cardiometabolic events and mortality, although most or all of this excess risk appears to be due to that conferred by the five risk factors contained within it [84].

Meta-analytic [20, 37, 40, 41] data and observational data from China [38] have confirmed a significantly increased risk of various diagnostic definitions of the metabolic syndrome associated with hypothyroidism vs. euthyroidism, although one of these studies found no significant increase in the criteria derived in China [20]. Increases in the risk of elevated obesity, blood pressure, and triglycerides, and of low HDL-C, drove the increased risk of metabolic syndrome in these analyses, consistent with the studies reviewed above. Conversely, people with the metabolic syndrome appear to be at increased risk of developing hypothyroidism [39].

#### Impact of LT4

LT4 has been shown to improve the various risk factors associated with the metabolic syndrome, as described elsewhere in this review and in uncontrolled studies in people who meet the diagnostic criteria for the metabolic syndrome [41, 85]. A study that involved withdrawal of LT4 treatment from subjects with thyroid cancer rendered athyreotic with radioactive iodine found that the resulting deterioration of cardiometabolic risk factors was more severe in patients with more metabolic syndrome components at baseline [86].

Hypothyroidism and metabolic syndrome: summary of clinical implications Few studies have been performed in populations with hypothyroidism and metabolic syndrome, especially involving the administration of LT4. The adverse effect of the metabolic syndrome on cardiometabolic outcomes can be explained largely by the effects of the individual risk factors. Accordingly, the evidence for exacerbation of metabolic syndrome components by hypothyroidism, and the available evidence for improved cardiometabolic risk factors with LT4, may be seen in the general context of improvements of these individual risk factors as described elsewhere in this review.

### Obesity

#### Impact of hypothyroidism

Obesity and excess adiposity are other well-described clinical consequences of hypothyroidism [1, 3]. A retrospective study from Spain showed that 76% of a population with hypothyroidism were overweight or obese, compared with 59% of people with hyperthyroidism [42]. Overweight/obesity is seen both in terms of increased body mass index (BMI) and waist circumference. While the TSH level correlates positively with BMI, the relationship between thyroid status and obesity is complex. Morbid obesity per se increases TSH, and bariatric surgery leads to a reduction in TSH without a change in the level of FT4 [87]. Increased adiposity may alter the set point of thyroid homeostasis, favouring a higher level of TSH for a given level of FT4, perhaps associated with increased secretion of leptin [87]. The observation that obese subjects have higher risk of a diagnosis of hypothyroidism [43] may reflect similar issues of confounding. However, an observational study found that obesity increased the risk of OH and thyroid autoimmunity, suggesting that other mechanisms may be at play [44].

#### Impact of LT4

In an observational study, treatment of people with OH with LT4 for 12 weeks resulted in improvements in weight, body mass index, waist circumference, and subcutaneous (but not visceral) fat [48]. However, a meta-analysis did not report a significant reduction in BMI in people with hypothyroidism receiving LT4 during treatment periods of up to one year [88]. Much of the weight gain in hypothyroidism often consists of excess salt and water [89], and people taking LT4 for hypothyroidism should not expect to lose substantial amounts of weight. For example, a retrospective evaluation of 95 patients starting LT4 reported a mean reduction in BMI of − 0.1 kg/m^2^, from a baseline mean value of 29.3 kg/m^2^, which indicates an overweight-to-obese population, on average [90]. Increased hunger during LT4 treatment has been implicated as one possible reason for limited weight loss during thyroid hormone replacement, according to one recent study [91].

Accordingly, thyroid hormone replacement with LT4 is not a rational strategy for achieving weight loss and changes in weight in this setting are unlikely to represent a mechanism for reducing cardiometabolic risk [92]. Overweight or obesity associated with excess fat, especially ectopic fat deposited in the liver, heart, pancreas and muscle, is a major driver of diabetes and adverse cardiometabolic outcomes and should be addressed in addition to the management of thyroid function [93].

Hypothyroidism and obesity: summary of clinical implications Hypothyroidism is associated with overweight/obesity, although this relationship is complex. Weight loss on LT4 is usually modest. Administration of LT4 should never be seen as a strategy for achieving weight loss.

## Hypothyroidism and vascular and cardiac function

**Fig. 2:**
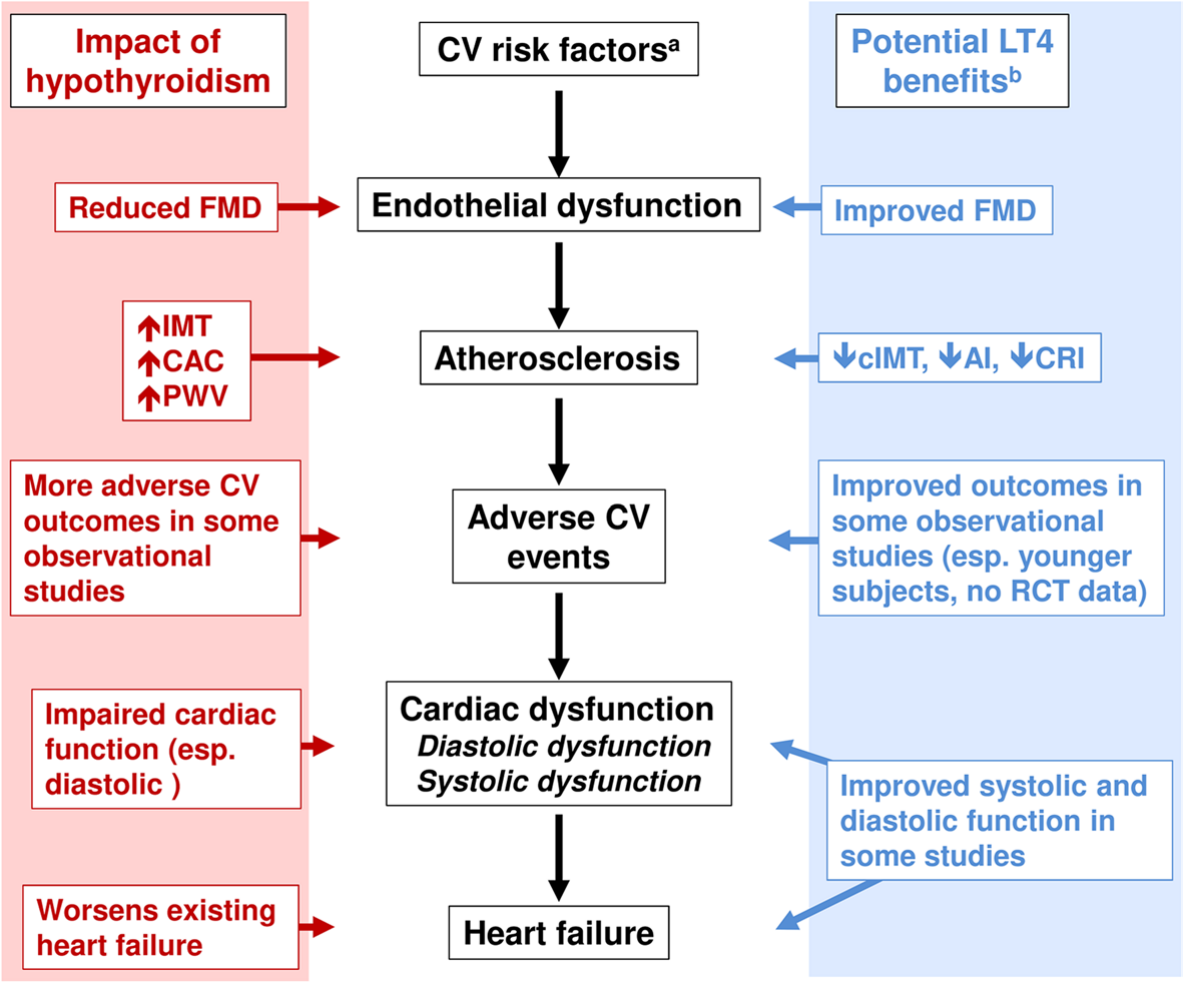
Overview of the effects of LT4 on cardiac structure and function in clinical studies ^a^See text and Fig. 1. ^b^These potential benefits have not been seen in all studies (see text). The order of progression of cardiovascular disease shown here is for illustrative purposes and does not always occur in this order (e.g. the onset of heart failure may not be preceded by an adverse cardiovascular event). AI: atherogenic index; CAC: coronary artery calcification; cIMT: carotid intima-media thickness; CRI: coronary risk index; CV: cardiovascular; FMD: flow-mediated vasodilatation; LT4: levothyroxine; PWV: pulse wave velocity; RCT: randomised, controlled trial. See text for further explanation and supporting references

Table 2 and Fig. 2 summarise the results of individual studies that reported the impact of hypothyroidism on the function of the heart and/or vasculature. The effects of LT4 on these parameters are summarised in Table 3 and Fig. 2.

**Table 2:** Overview of studies of effects of hypothyroidism on both vascular and cardiac structure as well as function and adverse cardiovascular outcomes

Ref	Type	Main findings
**Impaired vascular or cardiac function**		
[94]	Obs	A "quasi-experimental study" significantly associated SCH with impaired endothelial dysfunction
[95]	MA	A meta-analysis of 10 studies found decreased mean endothelial function in a population with SCH vs. euthyroid population
[96]	MA	A meta-analysis reported increased arterial stiffness (increased pulse wave velocity and diminished nitrate-induced vasodilatation) associated with SCH vs. a euthyroid population
[97]	Obs	Poor prognosis in patients with SCH, heart failure, TSH > 7 mIU/L and low triiodothyronine levels
[98]	Obs	Impaired diastolic cardiac function for people with SCH vs. control subjects
[99]	Obs	Impaired systolic and diastolic function observed in "mild SCH" (4.2 < TSH < 10.0 mIU/L) vs. controls
[100]	Obs	A magnetic resonance imaging technique revealed diffuse myocardial injuries in the hearts of people with SCH
**Atherosclerosis**		
[94]	Obs	No impact of SCH on cIMT
[94–96, 101]	MA	Meta-analyses reported increased mean cIMT in populations with SCH vs. euthyroid subjects
[102]	MA	Larger increase in cIMT in a population with SCH where TSH was > 10 mIU/L

Study types: *Obs* observational study, *MA* meta-analysis. Other abbreviations: *cIMT* carotid intima-media thickness, *SCH* subclinical hypothyroidism, *TSH* thyrotropin

**Table 3:** Overview of studies of effects of levothyroxine on vascular and cardiac structure and function,^a^ and clinical cardiovascular outcomes

Ref	Type	Main findings
**Vascular structure and function**		
[62]	RCT	Increased FT4 was the strongest multivariate predictor of improved endothelial function in a RCT of LT4 vs. placebo in a population with SCH
[65]	Obs	Improved endothelial function during LT4 treatment in a population of women with overt hypothyroidism
[48]	Obs	Improved indices of cardiovascular risk during treatment of patients with “clinical hypothyroidism” and TSH > 10 mIU/L with LT4 (atherogenic index, coronary risk index, Framingham risk score)
[94]	Obs	Administration of LT4 for 2 months to people with SCH resulted in a significant improvement in endothelial function, but with no effect on cIMT
[96]	Obs	Improved cIMT and reduced cardiac epicardial fat during treatment of people with SCH with LT4 vs. a control group or vs. baseline
[49, 68]	Obs	cIMT was reduced during treatment with LT4 in subjects with elevated TSH or a diagnosis of SCH
[73]	Obs	Administration of LT4 to a population with SCH reduced cIMT and epicardial fat
[101, 103]	MA	cIMT in patients with SCH who received LT4, especially for > 6 months
**Cardiac function**		
[104]	RCT	LT4 increased early diastolic velocity and early-to-late diastolic velocity and reversed increases in IVRT seen in SCH
[105]	RCT	LT4 reversed SCH-associated increases in pre-ejection/ejection time ratio and IVRT, peak transmitral flow velocity in late diastole (peak A) and cyclic variation index
[106]	RCT	LT4 improved IVRT, LVEF and CO in patients with chronic CHF
[107, 108]	RCT	LT4 increased LVEF, the shifted to right the slope of the end-systolic stress relation, reduced systemic vascular resistance and in increased CO in subjects with idiopathic dilated cardiomyopathy
[109]	RCT	No effect of LT4 on systolic or diastolic function in patients with mild SCH
[99]	Obs	LT4 increased LVEF and E/e' ratio and reduced myocardial performance index and lower global longitudinal strain in patients with new-onset mild SCH
[110]	Obs	12 months of LT4 improved multiple aspects of left and right ventricular structure dynamics in a population with SCH
[111]	Obs	12 months of LT4 improved PCr to ATP ratio in the hearts of people with SCH (improved myocardial bioenergetics)
[112, 113]	Obs	LT4 treatment was associated with increased risk of acute HF decompensation in people with HFpEF who were already receiving LT4 (the reason for treatment was unknown)
[114]	MA	LT4 improved (CO), left ventricular ejection fraction (LVEF), and the ratio of peak E velocity/peak A velocity in people with SCH
**Clinical cardiovascular outcomes**		
[115, 116]	Obs	The risk of CVD or mortality was increased in untreated, but not LT4-treated patients with SCH from a database in Scandinavia; both under treatment and over treatment increased the risk of these outcomes
[48]	Obs	LT4 reduced Framingham CHD risk score in people with “clinical hypothyroidism”
[117]	Obs	> 1 year of LT4 reversed the increased risk of new CHD in patients with Hashimoto's thyroiditis
[118, 119]	Obs	LT4 did not affect cardiovascular outcomes significantly in populations with SCH ± CVD
[120]	Obs	LT4 reduced risk of mortality or CHD in people with SCH aged < 65 y, but not in older patients
[121]	MA	LT4 reduced risk of all-cause or cardiovascular mortality or CHD in people with SCH aged < 65 y, but not in older patients

^a^See Table 2 for effects of overt hypothyroidism or SCH on cardiovascular structure and function. Study types: *RCT* randomised controlled trial, *Obs* observational study, *MA* meta-analysis. Other abbreviations: *CHD* coronary heart disease, *cIMT* carotid intima-media thickness, *CVD* cardiovascular disease, *CO* cardiac output, *HF* heart failure, *HFpEF* HF with preserved ejection fraction, *IVRT* isovolumetric relaxation time, *LVEF* left ventricular ejection fraction; SCH: subclinical hypothyroidism

### Impact of hypothyroidism

Impaired endothelium-derived vascular relaxation is an early event in the development of clinical cardiovascular disease and is associated strongly with an increased future risk of atherosclerosis and adverse cardiovascular outcomes. The presence of SCH was associated with reduced endothelial function, measured using flow-mediated vasodilatation, in a "quasi-experimental" study [94] and in a meta-analysis of 10 studies [95]. The same analysis reported a significant increase in mean carotid intima-media thickness (cIMT) in the SCH vs. euthyroid groups [95] that suggests strongly that SCH was associated with an increase in the overall burden of atherosclerosis. Other meta-analysis reported similar associations between endothelial function, cIMT progression or increased coronary artery calcification (another marker of atherosclerosis) [96, 101], especially where TSH was > 10 mIU/L [102]. One of these analyses also associated SCH with increased pulse wave velocity, a marker of increased arterial stiffness [96].

In the heart, hypothyroidism has been associated particularly with diastolic myocardial dysfunction and exacerbation of pre-existing heart failure [97, 98, 122]. Impairment of systolic cardiac function has also been described in populations with hypothyroidism and impairment of myocardial performance appears to correlate with the level of TSH [99, 100].

### Impact of LT4

Increased FT4 was the strongest predictor of improved endothelial function in a randomised, placebo-controlled trial in people with SCH [62]. Another observational study reported improved endothelial function after treatment of women with primary hypothyroidism with LT4 [65]. Treatment of people with OH with LT4 for 12 weeks resulted in improvements in several parameters relating to vascular function, such as atherogenic index and coronary risk index [48]. A meta-analysis demonstrated significant benefit for LT4 on cIMT whether LT4 was compared with a control group (placebo or no treatment) or before and after administration in the same patients [96]. Additional meta-analytic [101, 103] and observational [49, 68, 73] data have also documented reduced cIMT during treatment with LT4. Importantly, an observational study [96] and a meta-analysis [103] study showed that larger effects on cIMT were observed with longer durations of LT4 treatment (> 6 months), which would be consistent with a long-term improvement in cardiovascular risk. Not all studies reported reduced cIMT on LT4 treatment, however, including in a randomised, placebo-controlled trial [123].

Randomised trials have described improvements in regional systolic or diastolic cardiac performance with LT4 vs. control or placebo in populations with SCH [104, 105], or cardiac insufficiency without thyroid dysfunction [106–108]although not all trials have demonstrated such a clinical benefit [109]. These trials were generally small, however, and trials in more substantial populations are needed. Observational studies of relatively long duration (up to 1 year) [99, 110, 111] and a meta-analysis [114], have also demonstrated reversal of deficits in cardiac performance associated with hypothyroidism when patients treated with LT4 were compared with control groups.

A database study where LT4 treatment was associated with increased risk of acute cardiac decompensation in people with heart failure with preserved ejection fraction and normal TSH levels provides a note of caution in this area [112]. However, interpretation of this study is severely hampered by a lack of information on thyroid status and the reason for active treatment in the LT4 group [113].

Hypothyroidism and vascular/cardiac structure and function: summary of clinical implications Hypothyroidism-associated structural and functional abnormalities in the heart and vasculature are well described. These adverse changes are not unexpected, given the adverse impact of hypothyroidism on cardiovascular risk factors, described above. Indeed, it is well known that endothelial dysfunction and subclinical atherosclerosis (among other adverse changes) provide a clear path from individual cardiovascular risk factors to adverse clinical cardiovascular outcomes, which are described below. While signs of reversal of vascular and cardiac abnormalities during LT4 treatment are promising, more and larger randomised trials will be needed to realise this potential.

## Clinical cardiovascular outcomes

### Impact of hypothyroidism (Table 2)

A cohort of 3,021 people with SCH in South Korea was followed for cardiovascular outcomes for 12 years [124]. Compared with euthyroid control subjects, those with SCH and TSH in the highest quartile (> 6.7 mIU/L) were at increased risk of all-cause death (hazard ratio [HR] 2.12 [95%CI 1.27 to 3.56]) and cardiovascular events (HR 1.92 [95%CI 1.21 to 3.04). Higher risks were seen in subjects aged < 65 years with high CV risk at baseline (HRs (3.50 [95%CI 1.50 to 8.16] and 3.37 [95%CI 1.46 to 9.57], respectively). Other observational studies have reported higher rates of coronary heart disease or cardiovascular events in people with SCH vs. euthyroid status [33, 115, 116, 125, 126], including in populations with pre-existing coronary disease [127] or heart failure [128]. A large meta-analysis (35 studies) found a significant association between SCH and adverse cardiovascular outcomes in younger patients (< 65 years) and those with elevated cardiovascular risk [129]. Additional meta-analyses reported similar findings, with larger risks of coronary heart disease and associated mortality in people with SCH and TSH > 10 mIU/L [130, 131]. Higher FT4 and lower TSH, consistent with hyperthyroidism, were associated with increased risk of cardiovascular mortality in a large observational study [132], which may emphasise the importance of not over titrating LT4, thereby inducing a state of iatrogenic hyperthyroidism.

An observational study associated SCH with cerebrovascular disease [133], although a meta-analysis did not confirm this [134]. Finally, a Mendelian randomisation study significantly associated genetically predicted levels of FT4 with increased risk of coronary heart disease and stroke [14].

### Impact of LT4

A large database study from Scandinavia reported that both under- and over-treatment with LT4 were associated with increased risk of coronary heart disease, suggesting that appropriate TSH-guided management of thyroid homeostasis is more appropriate than prescribing LT4 per se [115, 116]. Long-term treatment of patients with autoimmune Hashimoto's thyroiditis with LT4 (> 1 year, vs. no treatment) reduced the risk of new coronary heart disease to the level seen in subjects without this condition [117]. In another study, treatment of 60 people with OH with LT4 for 12 weeks reduced the mean Framingham risk score, consistent with a reduction in the 10-year risk of adverse coronary outcomes [48]. Other observational studies did not report a significant effect of LT4 on cardiovascular outcomes in overall populations with SCH [118] or SCH with comorbid cardiovascular disease [119]. A subgroup analysis of one of these studies [118] and another study based on UK primary care data [120] reported a reduced risk of all-cause mortality and ischaemic heart disease events, respectively, in subjects with SCH aged < 65 years, with no significant effect in older subjects in either study. A large meta-analysis of studies in populations with SCH (7 studies, N = 21,055) reported that LT4 reduced all-cause and cardiovascular mortality only in patients aged < 65 years, with no significant effect in older patients or in the overall population [121].

The tendency for LT4 to be associated with reduced risk of adverse cardiovascular or mortality outcomes in younger subjects is intriguing. TSH levels tend to increase naturally with age without a corresponding increase in FT4, which has prompted speculation that SCH, in particular, may be over diagnosed in the elderly population [87]. Accordingly, it is possible that older populations with a diagnosis of hypothyroidism (especially SCH) will be enriched with subjects whose diagnosis of hypothyroidism may be open to question.

Hypothyroidism and clinical cardiovascular outcomes: summary of clinical implications We still await demonstration of improved clinical cardiovascular outcomes associated with thyroid hormone replacement with LT4 in a randomised, controlled trial. Real world evidence suggests such a benefit in younger people with SCH. Conversely, TSH-guided treatment of hypothyroidism with LT4 appears safe from a cardiovascular perspective, with no evidence of an increased risk of adverse cardiovascular outcomes.

## T3 treatment and cardiovascular risk factors

The sections of this review that have described the effects of thyroid hormone replacement on cardiometabolic risk factors and outcomes have focused on evaluations of LT4, as LT4 monotherapy is the guideline-driven standard of care for the management of hypothyroidism at this time [92, 135, 136]. The use of T3 within the personalised management of patients with OH appears to be common in real-life clinical practice [136], despite a lack of support for this approach in current guidelines for the management of hypothyroidism [135, 136].

LT4 acts as a prohormone for T3, which is the physiologically active thyroid hormone [137]. Observations of persistent hypothyroid-like symptoms in a substantial minority of people with hypothyroidism and well controlled TSH on LT4 monotherapy have stimulated interest in the therapeutic use of LT4 and T3 in combination, which could potentially better reflect the physiological situation. Clinical trials in this area in the early part of the current century showed little or no benefit for the LT4 + T3 combination vs. LT4 monotherapy [136]. However, subsequent discussion of design limitations of these trials has prompted a new generation of trials in subjects more likely to benefit compared with the relatively unselected hypothyroid populations recruited by the earlier trials, e.g. due to low T3 or the presence of polymorphisms in deiodinase enzymes that might suggest reduced availability of T3 at the level of target tissues for thyroid hormones [136]. A brief account of the effects of T3 on cardiometabolic risk factors and outcomes, focussing on more recent studies, will be included here for completeness.

A recent (2022) randomised trial found that T3 monotherapy improved quality of life measured using the validated, disease-specific ThyPro instrument, in women with residual hypothyroid-like symptoms on LT4 monotherapy or LT4 + T3, although there was no difference between groups for BP (average BP was not elevated in this population at baseline) [138]. Another randomised trial (2016) reported no difference between the effects of LT4 + T3 and T3 alone on BP or lipids [139].

With regard to cardiovascular function, a recent (2022) meta-analysis that analysed the effects of T3 treatment in subjects with low T3 reported an increase in cardiac index in adults undergoing cardiac surgery (high quality evidence), but no benefit in adults with heart failure or after myocardial infarction (low quality evidence) [140]. An acute increase in endothelial function was seen in an experimental study in human subjects [141]. Improved diastolic cardiac function was observed in a population without cardiovascular risk factors [142], and improved cardiac function has been seen in T3-treated patients with heart failure [143–145].

Currently, therefore, evidence for an additional effect of T3 on cardiometabolic parameters in people with hypothyroidism already treated with LT4 is scarce. This is perhaps unsurprising, as it is known already that intervention with LT4 improves risk factors such as dyslipidaemia and hypertension, and recent trials in this area have focussed more on the possibility of improving residual symptoms such as fatigue with additional T3. The suggestions of improved cardiac function with T3, described above, are intriguing. Further research is needed in populations with low T3 syndrome (a state of low T3 sometimes associated with cardiac conditions or surgery), which is associated with a poor clinical prognosis [146].

Importantly, adding T3 to LT4 therapy appears to be safe from a cardiometabolic perspective in people with hypothyroidism: there seems to be little evidence for an increased risk of the adverse cardiovascular sequelae of iatrogenic thyrotoxicosis, as long as routine measure to avoid over treatment with thyroid hormones are respected [147]. More recent evaluations of LT4 + T3 combination therapy have tended to use a lower ratio of LT4:T3 (about 14:1 or higher), compared with earlier studies (as high as about 1:5), which may mimic the euthyroid state more accurately and therefore maintain tolerability [147].

## Summary and conclusions

The studies summarised above show clearly that hypothyroidism (especially OH but also SCH), leads to adverse changes in cardiometabolic risk factors, such as dyslipidaemia, hypertension, obesity and hyperglycaemia. Effects on these risk factors may interact to exacerbate the situation further: for example, obesity worsens insulin resistance, especially in the setting of ectopic fat deposition in the liver, heart and muscle, which in turn tend to exacerbate sub-optimal blood glucose control, hypertension and hypertriglyceridaemia, among other effects (Fig. 1). Hypothyroidism is also associated with an increased likelihood of increased systemic vascular resistance, impaired diastolic dysfunction, decreased systolic function, and reduced preload. Damage to cardiovascular tissues caused by these adverse changes accumulates over time and leads to atherosclerosis, endothelial dysfunction, left ventricular hypertrophy and − ultimately − morbid cardiovascular events and death (Fig. 2). In addition, decreases in systolic and diastolic function in people with hypothyroidism contribute to impaired quality of life during exercise and even rest, contributing significantly to impaired quality of life and adding to the burden of disease [148].

The clinical evidence base suggests that TSH-optimised thyroid hormone replacement with LT4 may improve some cardiometabolic disease risk factors, particularly diastolic hypertension and dyslipidaemia. Real world evidence suggests that these benefits feed through to reduced manifestations of vascular and cardiac dysfunction, with suggestions of a reduced risk of adverse clinical cardiometabolic outcomes in younger people in SCH (Fig. 1). Randomised studies are needed to confirm this potential benefit of LT4 (and to provide further confirmation of the apparent good cardiovascular safety of this treatment) and to clarify the metabolic mechanisms by which alterations in thyroid homeostasis may influence the level of cardiometabolic risk in people with hypothyroidism.

## Data Availability

No datasets were generated or analysed during the current study.

## Abbreviations

DBP: Diastolic blood pressure
BP: Blood pressure(s)
SBP: Systolic blood pressure
LDL-C: Low-density lipoprotein cholesterol
HDL-C: High-density lipoprotein cholesterol
VLDL-C: Very low-density lipoprotein cholesterol
MAFLD: Metabolic-dysfunction-associated fatty liver disease
HMG-CoA: 3*-*Hydroxy-3-methylglutaryl coenzyme A
ApoB: Apolipoprotein B
BP: Blood pressure
SCH: Subclinical hypothyroidism
OH: Overt hypothyroidism
OR: Odds ratio
LT4: Levothyroxine
T4: Thyroxine
FT4: Free thyroxine
T3: Triiodothyronine
TSH: Thyrotropin (thyroid stimulating hormone)
BMI: Body mass index
cIMT: Carotid intima-media thickness
